# Mixed-biofilm natural transformation assay reveals the presence of staphylococci in human environments that can transfer SCC*mec* to *Staphylococcus aureus*

**DOI:** 10.1128/msphere.00442-25

**Published:** 2025-09-22

**Authors:** Mais Maree, Yuri Ushijima, Annisa Krama, Maaya Sasaki, Terumi Miyata, Masato Higashide, Le Thuy Thi Nguyen, Kazuya Morikawa

**Affiliations:** 1 Institute of Medicine, University of Tsukuba 38515, Tsukuba, Japan; 2 Graduate School of Comprehensive Human Sciences, University of Tsukuba 90521, Tsukuba, Japan; 3 Kotobiken Medical Laboratories, Inc., Tsukuba, Japan; 4 Biotechnology Center of Ho Chi Minh City 736684 , Ho Chi Minh City, Vietnam; University of Galway, Galway, Ireland

**Keywords:** *Staphylococcus*, MRSA, SCC*mec*, natural transformation, biofilm

## Abstract

Methicillin-resistant *Staphylococcus aureus* (MRSA) is an important pathogen that causes healthcare-, community-, and livestock-associated infections. The methicillin resistance gene *mecA* is embedded in the mobile genetic element termed Staphylococcal Cassette Chromosome (SCC*mec*). SCC*mec* is shared among staphylococci inhabiting human and animal hosts, which are recognized epidemiologically as the genetic reservoir of SCC*mec*. However, the ability of diverse methicillin-resistant staphylococci (MRS) to serve as SCC*mec* donors for *S. aureus* has not been tested experimentally. Here, we investigated the ability of 157 MRS isolates from pets, meat, livestock, and humans to transfer SCC*mec* to methicillin-sensitive *S. aureus* strains using a recently developed natural transformation protocol in mixed biofilms. We found that 25 out of 157 isolates were able to transfer SCC*mec* to *S. aureus*. The most effective donor species were *S. epidermidis* (~33% of the tested isolates), *S. felis* (40%), and *S. capitis* (30%). Isolates from meat and livestock (collected in Vietnam and Thailand) had lower transfer rates of SCC*mec* (5% and 3%, respectively), compared to human and pet isolates from Japan (35% and 25%, respectively). The SCC*mec* transfer depended on site-specific integration/excision mediated by an intact *attB* site, which is recognized by the SCC recombinase Ccr. Our study experimentally demonstrates the presence of SCC*mec* donors in our living environments, highlighting the importance of specific staphylococcal species.

**IMPORTANCE:**

How MRSA emerges has long been the pivotal question regarding the ever-increasing burden of antimicrobial resistance (AMR) issues for over half a century. Extensive research efforts in bacteriology, epidemiology, genome biology, and healthcare fields have led to the common understanding that SCC*mec* is transmitted among distinct staphylococcal species. However, global efforts to provide empirical evidence for intercellular SCC*mec* transmission have yielded limited results. We recently established the mixed-biofilm transformation assay to evaluate intercellular and interspecies SCC*mec* transmission. This novel assay system allows us to gain insight into the question “How MRSA emerges,” and here, we provide the first experimental results about the potential donor species and habitats. This is the first report to show the ability of staphylococci from distinct sources to transfer SCC to *S. aureus*. Moreover, the new finding of *S. felis* as an effective donor that is not commensal to humans reinforces the importance of the One Health concept.

## INTRODUCTION

The genus *Staphylococcus* consists of more than 80 species (1). They are common colonizers of the skin and mucosal surfaces of humans and warm-blooded animals and are frequently recovered from the environment (2), but some are opportunistic pathogens that cause infections in immunocompromised hosts (3). Coagulase-positive *S. aureus* is the most virulent, causing a broad range of human infections from superficial skin abscesses to life-threatening conditions such as meningitis, pneumonia, and sepsis.

The rapid emergence of antibiotic resistance is posing a serious global threat. The World Health Organization (WHO) predicts that antimicrobial resistance (AMR) will be the leading cause of death by 2050 (4), emphasizing the need for a thorough understanding of the emergence and spread of drug-resistant bacteria. Methicillin-resistant *S. aureus* (MRSA) is a major contributor to AMR among ESKAPE pathogens, causing substantial mortality and morbidity (5). MRSA is a leading cause of nosocomial infections, with prevalence rates in inpatients varying considerably by region: Japan 36%, Vietnam 73%, Thailand 12%, USA 45%, Norway 1% (6). MRSA has also been associated with community infections (community-associated MRSA [CA-MRSA]) and livestock infections (livestock-associated MRSA [LA-MRSA]) (7). New MRSA clones are constantly emerging in different regions (8), contributing to a substantial health and economic burden worldwide (7, 9).

β-Lactam antibiotics inhibit cell wall synthesis by irreversibly binding to the transpeptidase domain of penicillin-binding proteins (PBPs) (10). Resistance to these antibiotics is mediated by the *mecA* gene (or its homologs *mecB*, *mecC*, and *mec*D), encoding the low affinity PBP2’ protein (11–14). The *mecA* gene is located on the mobile genetic element Staphylococcal Cassette Chromosome *mec* (SCC*mec*), which is shared broadly among *Staphylococcus*, *Mammaliicoccus*, and *Macrococcus* species (8). At least 15 types of SCC*mec* have been reported, with types I–V (20–60 kb) being the most widespread in *S. aureus* (8, 15). Type I was identified in the first MRSA isolate from a UK hospital in 1961 (16), while the larger types II and III were later identified in clinical isolates from Japan and New Zealand (17). The smaller types IV and V (~20–24 kb) were first identified in the USA and Australia and are common in CA-MRSAs (18). SCC*mec* encodes the cassette chromosome recombinases (Ccr) that mediate its integration and excision in the *S. aureus* chromosome at a specific site (*attB*), located at the end of *orfX* (*rlmH*) gene (19, 20).

Coagulase-negative staphylococci (CoNS) are considered a major reservoir of SCC*mec* for *S. aureus*. Evolutionary precursors of the *mecA* gene and other components of SCC*mec* have been identified in animal-commensal CoNS such as *S. sciuri (M. sciuri*), *S. vitulinus*, *S. fleurettii*, and *M. caseolyticus* (2, 8, 21–24). A high diversity of SCC*mec* elements is present in CoNS from various habitats (2). SCC*mec* type IV in *S. epidermidis* shares a high sequence homology (98%–99%) with the one in MRSA (25), and while it was highly prevalent in *S. epidermidis* since the 1970s, it appeared in MRSA a decade later (26). In contrast, *S. haemolyticus* is suggested to be the reservoir of SCC*mec* type V (27).

While the clonal expansion of epidemic MRSA clones has played a role in the global spread of MRSA (28), evolutionary models predict *S. aureus* to have acquired SCC*mec* at least 20 independent times (29). *In vivo* horizontal gene transfer (HGT) of SCC*mec* from MR-CoNS to MSSA has been reported in a few cases, resulting in MRSA strain that shares the same genetic backbone of the MSSA strain in the same neonate (30–32). However, the ability of methicillin-resistant staphylococci (MRS) species, which inhabit diverse environments, to act as SCC*mec* donors for methicillin-sensitive *S. aureus* (MSSA), has not been investigated due to the lack of an appropriate experimental system to detect the SCC*mec* transfer.

Among the major HGT mechanisms in *S. aureus*, transduction and conjugation could mediate the transfer of short or fragmented SCC (33, 34). Natural transformation is another HGT mechanism that allows bacteria to uptake exogenous DNA via the expression of DNA uptake machinery encoded by the competence genes (*com* genes) (35). While the DNA uptake machinery is conserved among different genera (*Bacillus*, *Staphylococcus*, *Streptococcus*, etc.), the signaling cues and conditions controlling its expression vary (35). *S. aureus* develops competence in a subpopulation when grown in a specific complete synthetic medium (CS2), leading to the expression of the *comG* and *comE* operons, which are under direct transcriptional control of the alternative sigma factor SigH and the transcription factor ComK (36–38).

Biofilm growth conditions promote natural transformation in *S. aureus*, allowing the intra- and interspecies transfer of SCC*mec* elements (I–IVa) at frequencies of 10^−8^–10^−7^ (38). In a follow-up study, we further optimized the protocol with living donor cells in biofilm conditions with the efficiencies of SCC*mec* up to 10^−2^ (39). This protocol allows us to experimentally address the habitat and species of MRS that can act as SCC*mec* donors for *S. aureus*. Here, we present the results of our study involving 157 MRS strains collected from various animal environments and human sources.

## RESULTS

### Natural transformation in mixed biofilm can transfer SCC*mec* from MRSA to MSSA

**Fig 1:**
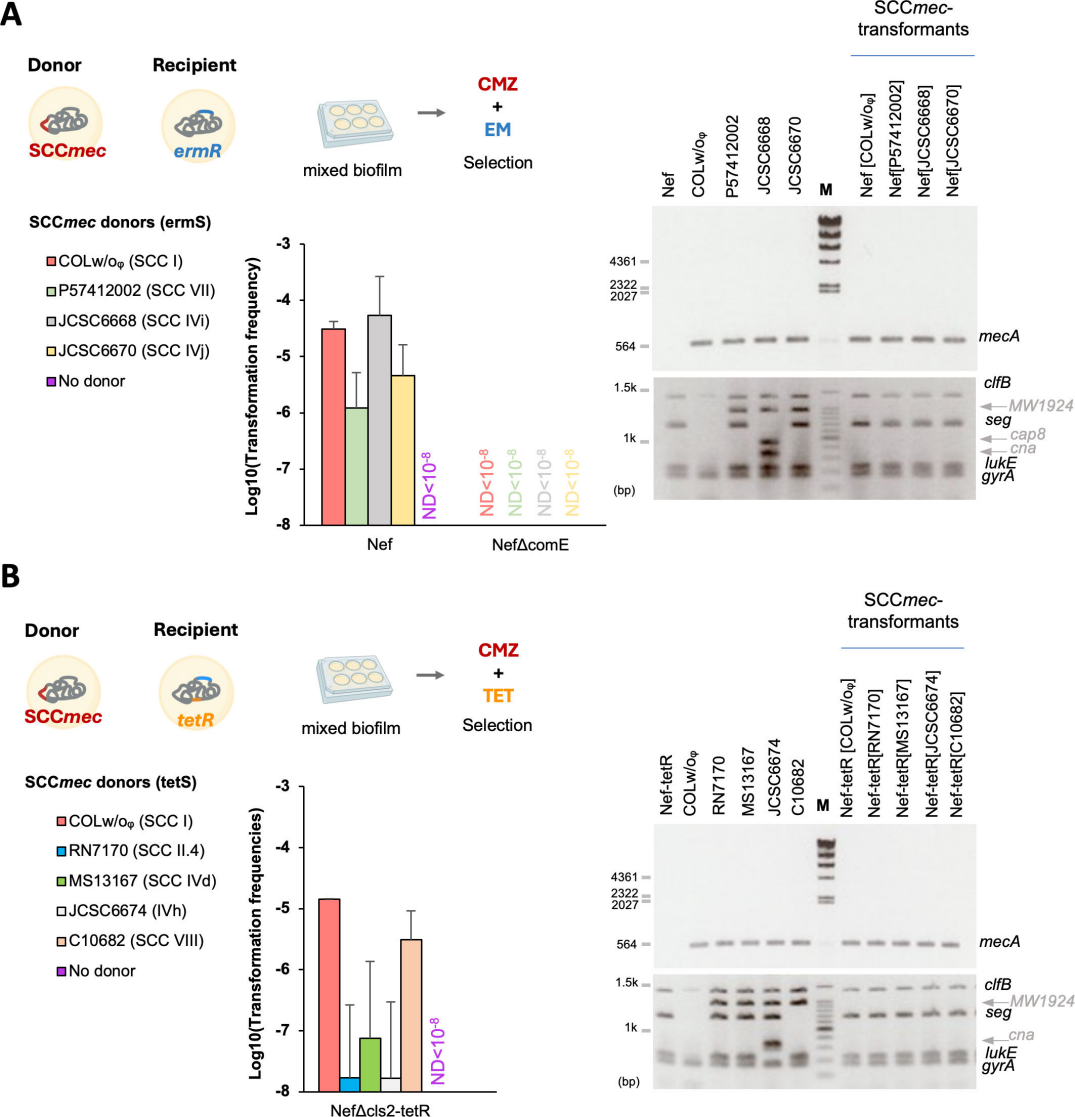
Transfer of diverse SCC*mec* elements in mixed biofilms. (**A and B**) Living SCC*mec-*donors were co-cultured with recipient cells (Nef, NefΔcomE, or NefΔcls2-tetR) at a 5,000:1 ratio. The cells were statically grown in CS2 medium at 37°C for 2 days. (**A**) Erythromycin sensitive donors (COLw/oᵩ, P57412002, JCSC6668, JCSC6670) were added to Nef or NefΔcomE recipients, and transformants were selected by erythromycin (EM) and cefmetazole (CMZ). ND, none detected. (**B**) Tetracycline-sensitive donors (RN7170, MS13167, JCSC6674, C10682) were added to NefΔcls2-tetR recipient, and transformants were selected by tetracycline (TET) and CMZ. For COLw/oᵩ donor, transformants were selected by EM and CMZ. The mean of *n* = 4 independent experiments is shown with SD. Some cartoon parts were created by BioRender. Right panels, The SCC*mec*-transformants of Nef and NefΔcls2-tetR have the mecA gene (upper gels) and share the same genetic backbone with the recipients (lower gels). Genomic DNA of donors and recipients was used as controls for PCR. M: λ-*Hin*dIII DNA ladder (upper gels) or 100 bp DNA ladder, Takara (lower gels: largest signal is 1.5 kbp and others are 100 bp ladder with the major signal at 1 kbp).

We previously demonstrated the transfer of SCC*mec* types I and IVa in mixed biofilms using live donor cells with a high donor-to-recipient ratio of 5,000:1 (39). For example, MRSA COLw/oφ (COL derivative that lacks conjugative genes and the lysogenized phage but carries SCC*mec* type I, Table S5) can transfer SCC*mec* at a frequency of 10^−4^ to 10^−5^ to the recipient MSSA Nef (N315 derivative that lacks conjugative genes, prophages, and SCC*mec*) (Fig. 1A). Using this protocol, we first confirmed that additional SCC*mec* types (IVd, IVh, IVi, IVj, VII, VIII, II.4) from MRSA donors can be transferred to MSSA recipients of Nef or Nef△cls2-tetR (Nef derivative carrying a chromosomal *tet*R gene) (Fig. 1A and B). All the tested MRSA could serve as SCC*mec* donors at mean frequencies ranging from 10^−8^ to 10^−4^. No transfer happened in the negative control recipient strain NefΔcomE, which lacks the *comE* operon encoding the DNA incorporation machinery. The presence of the *mecA* genes was confirmed by PCR in all the transformants, and they shared the same genetic backbone as their recipients in multiplex PCR test (Fig. 1, right panels). To provide sequence-based confirmation of SCC*mec* transfer and chromosomal integration, we performed whole-genome sequencing on several transformants: Nef[JCSC6668], Nef-tetR[MS13167], Nef-tetR[C10682], Nef[P57412002], Nef[JCSC6670], and Nef-tetR[COLw/oϕ]. The assembled contigs were analyzed using SCCmecFinder (40), which confirmed the presence of integrated SCC*mec* elements in all genomes (Table S6). For Nef-teR[COLw/oϕ] and Nef[JCSC6670], the entire SCC*mec* element was identified on a single contig, enabling clear visualization of the cassette via genome alignment (Fig. S1).

### Characteristics of MRS that can transfer SCC*mec* to *S. aureus*

We selected 157 MRS strains from our collection isolated from humans (Japan), pets (Japan), livestock (Vietnam), and meat samples (Vietnam and Thailand) (Y. Ushijima, L. T. T. Nguyen, and A. Krama, unpublished data). These strains are susceptible to at least one of the antibiotics (erythromycin, tetracycline, chloramphenicol, or kanamycin), allowing them to be distinguished from the *S. aureus* recipient strains during transformation assays. All isolates harbored the *mecA* gene and carried diverse SCC*mec* elements including non-typeable variants (Tables S1 to S4).

MSSA recipients included Nef, Nef derivative strains with distinct resistance markers (Nef△cls2-tetR, Nef-pRIT5h, Nef-pMKcomGgfp) and clinical isolates (9s-ermR, E6, 98s) (39) (Table 1). Recipients Nef△comE and 9s△comE-ermR were used as negative controls. SCC*mec* transformants were selected using cefmetazole, alongside another antibiotic based on donor susceptibility. The transformants were confirmed by their ability to grow in the presence of cefmetazole on replica and by their identical genetic backbone with the recipient (39). Out of 157 isolates, 25 (16%) were able to transfer SCC*mec* to at least one MSSA recipient in one or more independent experiments. Transformation frequencies by the successful donors are shown in Table 1. None of the MRS susceptible to chloramphenicol or kanamycin (Tables S1, S3 and S4) could serve as a donor in at least two independent experiments. While there was a geographic bias in the sampling (human and pet samples were from Japan, while meat and livestock samples were from Vietnam and Thailand), the following tendencies were observed.

**TABLE 1:** Transformation frequencies of MRS donors^*a*^

*mecA*+ donor	SCC*mec* type	Species	Resistance marker(s)^*b*^	MSSA recipient: resistance marker(s)								
				Nef: *ermR*	NefΔcomE: *ermR*	9s-ermR: *ermR*	9sΔcomE-ermR: *ermR*	E6: *ermR*	98s: *ermR, tetR*	NefΔcls 2-tetR: *ermR, tetR*	Nef-pRIT 5H: *cmR*	Nef-pMKcom Ggfp: *kmR*
COLw/oᵩ	I	*S. aureus*	*ermS, tetR, cmS, kmS*	**2.0 × 10^−4^** ± 2 × 10^−4^ (*n* = 3)	ND (*n* = 3)	**1.0 × 10^−7^** ± 1 × 10^−7^ (*n* = 2), ND (*n* = 1)	ND (*n* = 3)	**9.0 × 10^−7^** ± 1 × 10^−6^ (*n* = 3)	**3.0 × 10^−6^** ± 5 × 10^−6^ (*n* = 3)	**4.0 × 10^−5^** ± 2 × 10^−5^ (*n* = 3)	**3 × 10^−4^** ± 6.9 × 10^−5^ (*n* = 2)	**8.0 × 10^−6^** ± 8 × 10^−6^ (*n* = 2)
J-P4	IVa/IVc	*S. epidermidis*	*ermS*	ND (*n* = 3)	ND (*n* = 3)	ND (*n* = 3)	ND (*n* = 3)	**2 × 10^−8^** ± 2 × 10^−7^ (*n* = 2), ND (*n* = 1)	**4 × 10^−8^** ± 5 × 10^−8^ (*n* = 2), ND (*n* = 1)			
J-P10	V/IVa	*S. epidermidis*	*ermS*	**1.3 × 10^−6^** ± 1.7 × 10^−6^ (*n* = 2), ND (*n* = 1)	ND (*n* = 3)	ND (*n* = 3)	ND (*n* = 3)	ND (*n* = 3)	**2 × 10^−8^** ± 3 × 10^−8^ (*n* = 2), ND (*n* = 1)			
J-P15	IVc/IVb	*S. felis*	*ermS*	**1.3 × 10^−6^** (*n* = 1), ND (*n* = 2)	ND (*n* = 3)	ND (*n* = 3)	ND (*n* = 3)	ND (*n* = 3)	ND (*n* = 3)			
J-P20	III	*S. cohnii*	*ermS*	ND (*n* = 3)	ND (*n* = 3)	ND (*n* = 3)	ND (*n* = 3)	**3 × 10^−8^** ± 5 × 10^−8^ (*n* = 2), ND (*n* = 1)	ND (*n* = 3)			
J-P22	Unknown	*S. lugdunensis*	*ermS*	**6 × 10^−8^** ± 9 × 10^−8^ (*n* = 2), ND (*n* = 1)	ND (*n* = 3)	ND (*n* = 3)	ND (*n* = 3)	ND (*n* = 3)	ND (*n* = 3)			
J-P24	Unknown	*S. felis*	*tetS*						ND (*n* = 2)	**7.3 × 10^−7^** (*n* = 1), ND (*n* = 1)		
J-P30	Unknown	*S. felis*	*tetS*						**5.0 × 10^−8^** (*n* = 1), ND (*n* = 1)	ND (*n* = 2)		
J-P31	IVb	*S. felis*	*ermS*	ND (*n* = 3)	ND (*n* = 3)	ND (*n* = 3)	ND (*n* = 3)	**3 × 10^−6^** ± 4 × 10^−6^ (*n* = 2), ND (*n* = 1)	ND (*n* = 3)			
J-P32	Unknown	*S. lugdunensis*	*ermS*	ND (*n* = 3)	ND (*n* = 3)	ND (*n* = 3)	ND (*n* = 3)	**3 ×** 10–6 ± 4 × 10^−6^ (*n* = 2), ND (*n* = 1)	ND (*n* = 3)			
J-P34	III	*S. nepalensis*	*tetS*						ND (*n* = 2)	**2.0 × 10^−6^** (*n* = 1), ND (*n* = 1)		
J-H2	II/V	*S. epidermidis*	*ermS*	ND (*n* = 3)	ND (*n* = 3)	ND (*n* = 3)	ND (*n* = 3)	ND (*n* = 3)	**2.3 × 10^−7^** (*n* = 1), ND (*n* = 2)			
J-H9	I/IVa	*S. epidermidis*	*ermS*	**2 × 10^−8^** (*n* = 1), ND (*n* = 2)	ND (*n* = 3)	ND (*n* = 3)	ND (*n* = 3)	ND (*n* = 3)	ND (*n* = 3)			
J-H10	III/V	*S. epidermidis*	*tetS*						**8.3 × 10^−8^** (*n* = 1), ND (*n* = 1)	**1.5 × 10^−7^** (*n* = 1), ND (*n* = 1)		
J-H16	IVb	*S. capitis*	*ermS*	ND (*n* = 3)	ND (*n* = 3)	ND (*n* = 3)	ND (*n* = 3)	**2 × 10^−8^** (*n* = 1), ND (*n* = 2)	ND (*n* = 3)			
J-H19	I/IVa	*S. epidermidis*	*tetS*						**1.7 × 10^−7^** (*n* = 1), ND (*n* = 1)	ND (*n* = 2)		
J-H25	Unknown	*S. capitis*	*ermS*	**1.6 × 10^−6^** ± 1.6 × 10^−6^ (*n* = 2), ND (*n* = 1)	ND (*n* = 3)	**1.3 × 10^−7^** (*n* = 1), ND (*n* = 2)	ND (*n* = 3)	ND (*n* = 3)	ND (*n* = 3)			
J-H26	IVa	*S. epidermidis*	*ermS*	**1.8 × 10^−7^** (*n* = 1), ND (*n* = 2)	ND (*n* = 3)	ND (*n* = 3)	ND (*n* = 3)	**3.1 × 10^−7^** (*n* = 1), ND (*n* = 2)	ND (*n* = 3)			
J-H30	IVa	*S. epidermidis*	*ermS*	**4.5 × 10^−6^** (*n* = 1), ND (*n* = 2)	ND (*n* = 3)	ND (*n* = 3)	ND (*n* = 3)	ND (*n* = 3)	ND (*n* = 3)			
J-H31	IVa/I	*S. epidermidis*	*ermS*	**2.6 × 10^−6^** ± 2.7 × 10^−6^ (*n* = 2), ND (*n* = 1)	ND (*n* = 3)	**6.0 × 10^−7^** (*n* = 1), ND (*n* = 2)	ND (*n* = 3)	ND (*n* = 3)	**1.0 × 10^−6^** ± 1.4 × 10^−6^ (*n* = 2), ND (*n* = 1)			
J-H35	I	*S. caprae*	*ermS*	**1.8 × 10^−7^** (*n* = 1), ND (*n* = 2)	ND (*n* = 3)	ND (*n* = 3)	ND (*n* = 3)	ND (*n* = 3)	ND (*n* = 3)			
J-H36	IVa	*S. epidermidis*	*ermS*	ND (*n* = 3)	ND (*n* = 3)	ND (*n* = 3)	ND (*n* = 3)	ND (*n* = 3)	**6.3 × 10^−8^** (*n* = 1), ND (*n* = 2)			
J-H37	I	*S. capitis*	*ermS*	ND (*n* = 3)	ND (*n* = 3)	ND (*n* = 3)	ND (*n* = 3)	ND (*n* = 3)	**8.8 × 10^−8^** ± 1.6 × 10^−8^ (*n* = 2), ND (*n* = 1)			
V-M9	Unknown	*S. sciuri*	*ermS*	ND (*n* = 3)	ND (*n* = 3)	ND (*n* = 3)	ND (*n* = 3)	ND (*n* = 3)	**9.0 × 10^−7^** (*n* = 1), ND (*n* = 2)			
V-M16	Unknown	*S. cohnii*	*tetS*						ND (*n* = 3)	**1.5 × 10^−7^** (*n* = 1), ND (*n* = 2)		
V-L10	Unknown	*S. gallinarum*	*ermS*	ND (*n* = 3)	ND (*n* = 3)	ND (*n* = 3)	ND (*n* = 3)	**3.3 × 10^−7^** (*n* = 1), ND (*n* = 2)	ND (*n* = 3)			

^*a*^ The average transformation frequencies (bold) ± SD are shown. *n* indicates the number of independent experiments. ND, none detected.

^*b*^ Resistance marker indicates the antibiotic to which the donor is susceptible and the recipient is resistant. This allows for the elimination of the donor when selecting transformants with CMZ.

**Fig 2:**
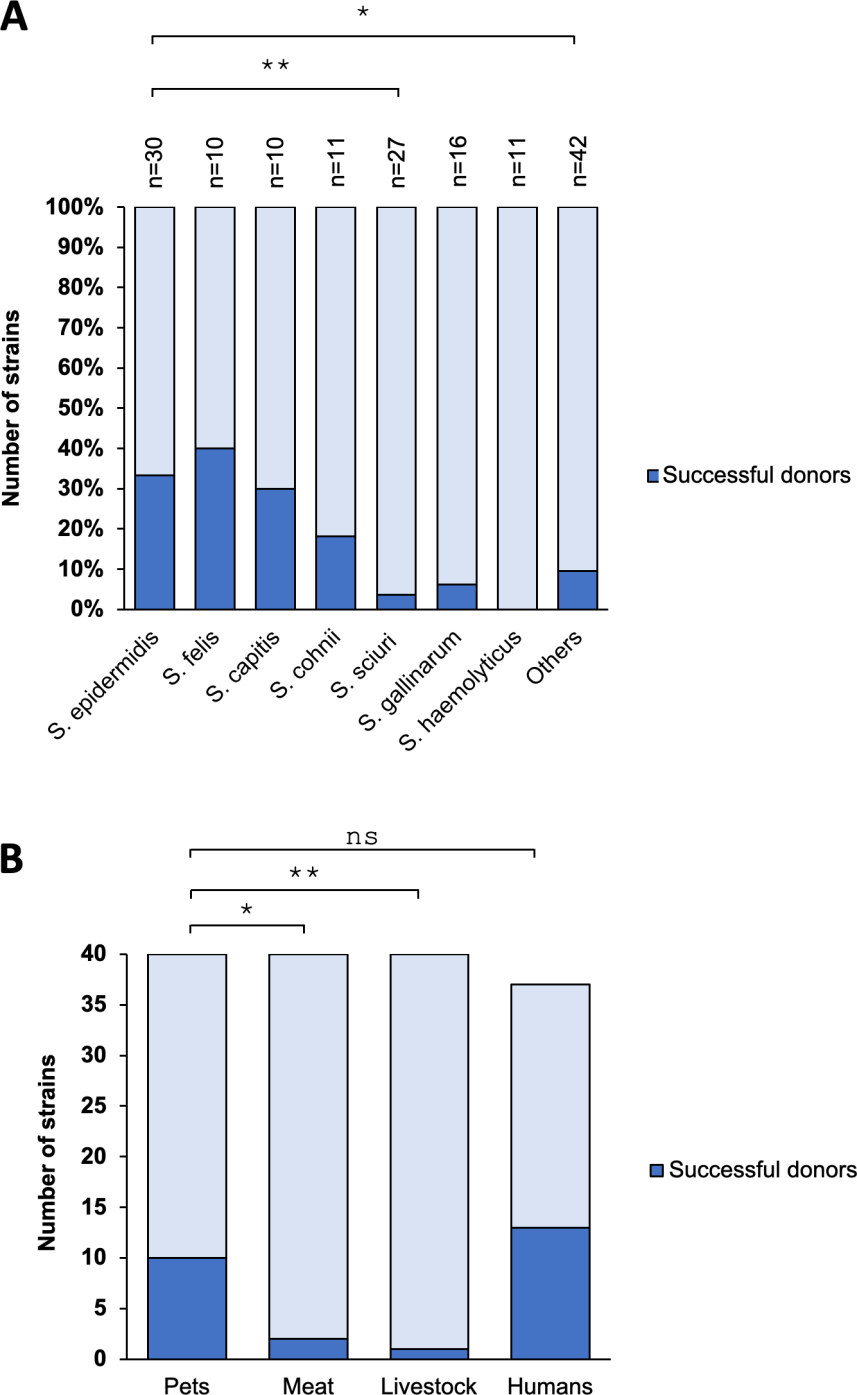
MRS from different sources can serve as SCC*mec* donors. (**A**) The percentages of the strains capable of serving as SCC*mec* donors (dark blue bars) from different species are shown. *n* represents the sample number. (**B**) The total number of strains that could serve as donors (dark blue bars) or not (light blue bars) is shown for the samples derived from pets, meat, livestock, and human sources. Statistical significance (**A and B**) was determined by Fisher’s exact test. *P < 0.05, **P < 0.01, ns, not significant.

There was a notable species dependency in their ability to transfer SCC*mec*. Among *S. epidermidis* strains, ~33% (10 out of 30, isolated from humans and pets) could serve as donors. Similarly, 40% of *S. felis* strains (4 out of 10, found only in pets) and 30% of *S. capitis* (3 out of 10, from humans) were also capable of serving as donors (Fig. 2A). In contrast, 18% of *S. cohnii* (2 out of 11, from pets and meat), 4% of *S. sciuri* strains (1 out of 27, from meat), and 6% of *S. gallinarum* (1 out of 16, from livestock) were able to transfer SCC. No strains of *S. haemolyticus* (0 out of 11) could transfer SCC*mec*. A subset of other species ~10% (4 out of 42, from pets and humans), including *S. lugdunensis* (2 out of 3), *S. caprae* (1 out of 5), *S. nepalensis* (1 out of 3), could also transfer SCC*mec*. These findings suggest that specific CoNS species, particularly *S. epidermidis, S. felis,* and *S. capitis,* play a critical role as SCC*mec* donors for *S. aureus* (Fig. 2A).

Among MRS isolates from pets and humans, 10 out of 40 (~25%) and 13 out of 37 (35%) could serve as SCC*mec* donors, respectively (Fig. 2B). In clear contrast, only 2 out of 40 strains from meat (~5%) and 1 out of 40 tested strains from livestock (~3%) could serve as donors, significantly lower than those from pets.

### SCC*mec* transfer relies on *attB* sequence

**Fig 3:**
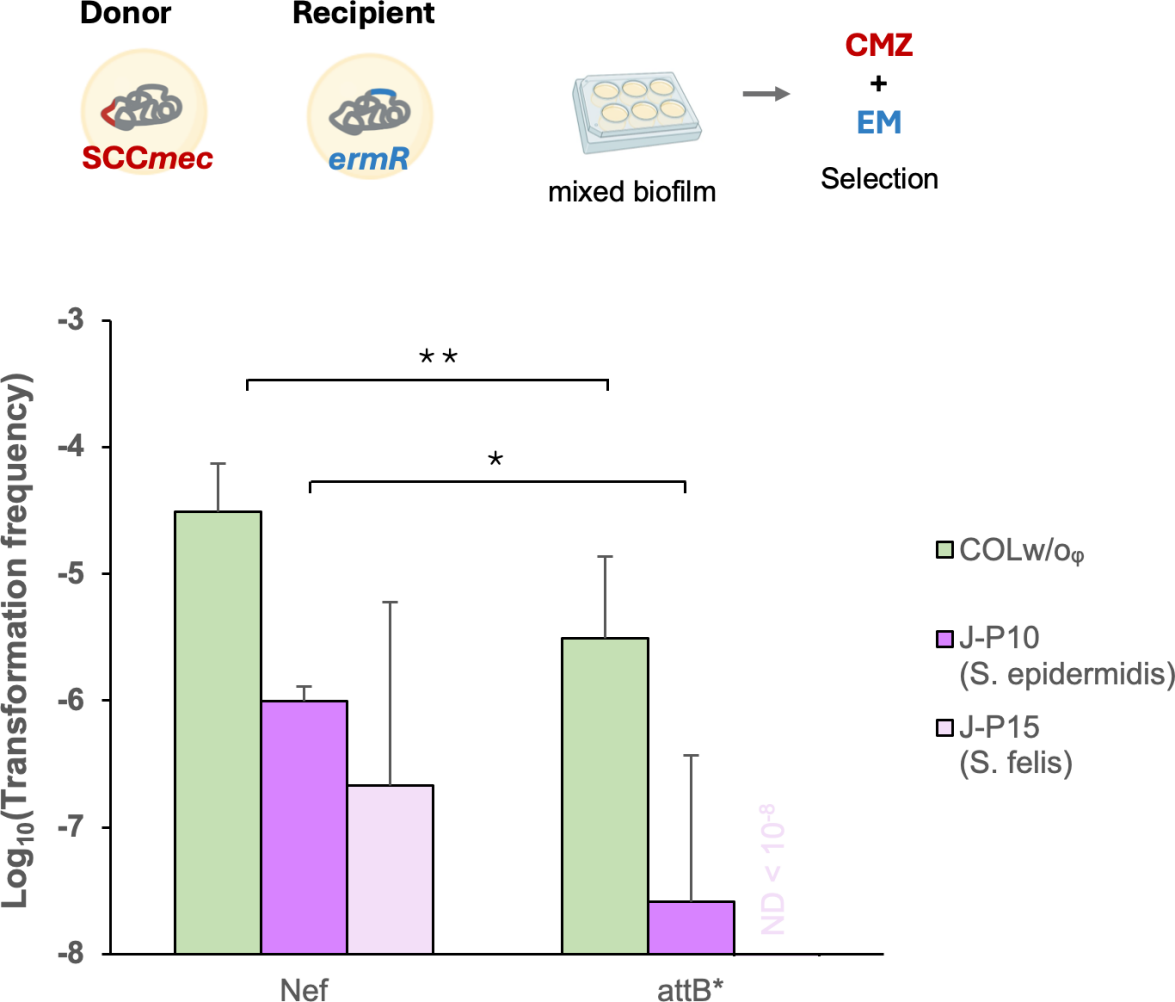
Dependency of SCC*mec* transfer on *attB* sequence. Living donor (COLw/oᵩ, J-P10, J-P15) cells were added to recipient cells (Nef or attB*) in a 5,000:1 ratio. The cells were statically grown in CS2 for 2 days at 37°C. Transformants were selected by erythromycin (EM) and cefmetazole (CMZ). The mean of *n* = 4–5 independent experiments is shown with SD. ND, none detected. Statistical significance was determined by a Student’s *t*-test. *P < 0.05, **P < 0.01.

Integration and excision of SCC*mec* occurs at the *attB* site of the *orfX* gene and is mediated by the cassette chromosome recombinases (Ccr) (8). Previously, we observed that transfer of SCC*mec* type I from MRSA donor (COLw/oφ) was dependent on the Ccr-attB system, by deleting the *ccrAB* genes from the donor and using the attB* recipient mutant (39). To investigate whether the transfer of SCC*mec* from the CoNS isolates also depends on the *attB* sequence, we tested the ability of SCC*mec*-donors J-P10 (*S. epidermidis*) and J-P15 (*S. felis*) from pets to transfer SCC to Nef and its attB* mutant recipients (Fig. 3). SCC*mec* transfer from all donors occurred at lower frequencies in attB* mutants, with ~10-fold reduction using COLw/oφ donor, ~13-fold reduction using J-P10 donor, and no transfer detected using J-P15 donor. This reinforces the importance of the *attB* sequence in SCC*mec* transfer in mixed biofilms.

## DISCUSSION

Methicillin resistance is conferred by SCC*mec*, a mobile genetic element that is shared among *Staphylococcus*, *Mammaliicoccus*, and *Macrococcus* species (8). This experimental study presents the first robust evidence of the presence of SCC*mec* donors for *S. aureus* in our environment.

Transformation of *S. aureus* was first reported by our group (37), but the original protocol using heat-killed donor cells in planktonic culture was extremely low, and the transformation was nearly undetectable in unmodified strains. In 2022, we reported that biofilm growth conditions improve the efficiency (38). This protocol gave rigid evidence that SCC*mec* transfer is mediated by natural transformation, but the efficiency was still low: 10^−8^–10^−7^, and other groups had difficulty in detecting the SCC*mec* transformation (41, 42). Recognizing this limitation, we further optimized the protocol to enhance SCC*mec* transfer efficiency (39). The updated method, published in 2024, uses living donor cells and mixed co-culture biofilm conditions, which significantly improve the efficiency and likelihood of successful transformation. The current study employs this improved protocol, which we believe is a key factor enabling the detection of SCC*mec* transfer in laboratory settings.

Among the tested MRS species, *S. epidermidis* (mostly carrying SCC*mec* IVa), along with *S. felis* and *S. capitis*, was the most efficient at transferring methicillin resistance to *S. aureus* (Fig. 2A). This corroborates previous studies identifying *S. epidermidis* as a reservoir of SCC*mec* type IV, which has been highly prevalent in *S. epidermidis* over a decade before discovery in MRSA (25, 26). The transfer of this element was likely facilitated by the ecological overlap among the species in the human niche (43). Notably, our study also identified *S. felis*, typically found in cats, as an efficient SCC*mec* donor for *S. aureus*, further supporting the role of companion animals in the spread of resistance. We tested only three *S*. *pseudintermedius* isolates (a dog commensal), and its role as a potential SCC*mec* donor remains unclear.

Despite evidence suggesting that SCC*mec* has originated and assembled in CoNS species such as *S. sciuri* and *S. fleuretti* (2), transfer of SCC*mec* from these species was rarely successful in the present study (1 donor *S. sciuri* strain out of 27), suggesting that SCC*mec* transfer to *S. aureus* has preferentially occurred via other species. The dissemination of SCC*mec* among CoNS before its transfer to *S. aureus* could have been a necessary step for its divergence, given the high plasticity and recombination rate in CoNS genomes (2, 44).

Our study does not delineate why certain species or strains are more successful SCC*mec* donors. We found no clear correlation between the SCC*mec* type or size and the transferability from different species (Table 1). Specific genetic factors, phylogeny, or physiological traits may influence the ability to transfer SCC*mec*. Factors such as growth in transformation conditions, communication with recipient *S. aureus* (45), ability to release DNA (39), and efficient excision/integration of SCC*mec* elements, which is influenced by the sequences flanking *orfX* region (46), could play a role.

The higher prevalence of SCC*mec* donors among isolates from humans and pets (35% and 25%, respectively), compared to only 5% in meat and 3% in livestock (Fig. 2), implies that close human-pet interactions are a critical factor in mediating the emergence of methicillin resistance. Pets can also be reservoirs of important drug-resistant pathogens such as MRSA, vancomycin-resistant *Enterococcus* (VRE), and multidrug-resistant *Salmonella typhimurium* DT104 (47). The shared living environments and intimate contact between pets and their owners facilitate the exchange of microflora and antibiotic-resistance genes among pathogens (48). Drug-resistant Gram-negative pathogens, such as *Enterobacteriaceae* and *Pasteurella multocida*, are transmitted from pets to their owners (49). Additionally, several studies reported the transmission of *S. aureus* between humans and their companion animals and, in rarer cases, the transmission of CoNS such as *S. pseudintermedius* and *S. felis*, including methicillin-resistant strains, from dogs and cats to humans (1, 50–53). This cross-species transmission can promote polymicrobial biofilm formation and create conducive environments for genetic exchange and dissemination of AMR.

The wide use of β-lactams in veterinary medicine and animal feed has been a key driver for the emergence of methicillin resistance (2). MRS species are frequently isolated from pets and livestock (54–56), with one study reporting multidrug resistance among 77.1% of isolated *Staphylococcus* species from pet dogs (57). The current use of phenicols and lincosamides in treating pet infections may exert selective pressure for the emergence of linezolid-resistant staphylococci through the dissemination of *cfr*-carrying plasmids, as has been observed in livestock populations (58, 59). Prudent use of antibiotics, combined with enhanced surveillance and development of strategies to limit HGT (60), is crucial to mitigate the spread of drug-resistant pathogens.

It might be noteworthy that successful donors among the meat isolates (2 out of 21) were from Vietnam, a region with a higher prevalence of MRSA, whereas no donors were identified from the less endemic region of Thailand (0 out of 19). This underscores the potential impact of regional differences in the usage of antibiotics and healthcare practices on the spread of AMR. Expanding the sample size and including a broader range of species, hosts, and geographical regions will be important in future studies to further clarify the environmental factors driving MRSA emergence.

In summary, our study presents experimental evidence for SCC*mec* transfer from CoNS of various hosts and species to *S. aureus*, providing crucial insights into the environments that contribute to MRSA emergence and dissemination. The transfer was most successful from species associated with humans and companion animals in mixed biofilms, emphasizing their role in promoting HGT and disseminating AMR (61). Our findings highlight the intricate connections between humans, animals, and their shared environment in disseminating antibiotic resistance genes, supporting the urgent need for One Health approach in combating the global AMR crisis.

## MATERIALS AND METHODS

### Bacterial strains, primers, and media

The *S. aureus* strains and primers used in this study are shown in Table S5. The 157 MRS isolates were selected from our collection (Ushijima et al., in preparation) and used in this study Tables S1 to S4. Clinical human and pet samples were retrieved from Kotobiken Inc. in the Kanto area of Japan between 2017 and 2024. Meat isolates were collected from markets in Thailand and Vietnam between 2022 and 2023, and isolates from livestock and their environments were obtained from farms in Vietnam in 2023 by swab cultures that were streaked on mannitol agar plates supplemented with 4 µg/mL cefoxitin to isolate the MRS species. Species were identified by MALDI Biotyper (Bruker-Daltonics, Inc) according to the manufacturer’s instruction. In brief, colonies were mixed with the matrix (HCCA : a-Cyano-4-hydroxycinnamic) and submitted to the mass-spectrometer miroflex LT/SH (Bruker). The obtained mass spectrometry peak pattern was compared with the database BDAL10833 (Bruker).

Staphylococci were routinely grown in TSB at 37°C with shaking (180 rpm). For transformation assays, CS2 (complete synthetic medium) was used. Where required for selection, the growth medium was supplemented with chloramphenicol (12.5 µg/mL), tetracycline (5 µg/mL), kanamycin (100 µg/mL), cefoxitin (4 µg/mL), cefmetazole (4 µg/mL), or erythromycin (16 µg/mL).

### Characterization of MRS

The presence of the *mecA* gene was tested by PCR using the primers mecAF and mecAR (38). SCC*mec* typing was performed based on the method and primers described by Ghaznavi-Rad et al. (62). Multiplex PCR was performed on extracted genomes using the QIAGEN multiplex PCR kit following the manufacturer’s instructions. PCR was carried out under the following conditions: initial denaturation at 95°C for 15 min, followed by 45 cycles of 94°C for 30 s, 60°C for 90 s, and 72°C for 90 s, with a final extension at 72°C for 10 min. PCR products were analyzed on a 2% agarose gel stained with ethidium bromide and visualized under UV light.

### Antimicrobial susceptibility testing

Disk-diffusion testing was performed based on the CLSI standards using the colony-suspension method as previously described (38). Briefly, isolated colonies of the tested species were inoculated in 0.85% NaCl for achieving turbidity equivalent to 0.5 McFarland standard. The inocula were cultured on Mueller–Hinton agar plates and the antibiotic disks of oxacillin (1  µg) and cefoxitin (30  µg) (KB disks, Eiken Chemical) were used for testing. The zones of inhibition were determined following incubation at 35°C for 18 h.

To test the ability of MRS to grow in erythromycin (16 µg/mL), tetracycline (5 µg/mL), chloramphenicol (12.5 µg/mL), or kanamycin (50 or 100 µg/mL), overnight cultures in TSB were diluted 1:1,000 and inoculated into fresh TSB medium containing any of these antibiotics. Resistance was determined by the ability to grow with the supplemented antibiotic following incubation at 37°C for up to 24 h.

### Natural transformation assay using living donor

Natural transformation assays using a living donor were conducted as previously reported (39). Seventy-five microliters of donor overnight culture (∼10^9^ CFU/mL in TSB) was washed once in CS2 medium (composition described in our previous study) (37). Overnight cultures of the recipient strain in TSB were also washed and diluted 200-fold in CS2 medium. A mixture of 75 µL washed donor, and 3 µL of the diluted recipient was added to each well of a polystyrene 6-well plate, achieving an approximate donor-to-recipient ratio of 5,000:1. The final volume in each well was adjusted to 1.5 mL with CS2 medium. The plate was incubated statically for 2 days at 37°C, with the medium refreshed after 24 h. The biofilm was then collected by extensive pipetting and poured into melted BHI agar supplemented with appropriate antibiotics. To select for transformants originating from antibiotic-resistant recipients while excluding susceptible donors, erythromycin (16 µg/mL), tetracycline (5 µg/mL), chloramphenicol (12.5 µg/mL), or kanamycin (100 µg/mL) was used. Cefmetazole (4 µg/mL) was added to select for the *mecA* gene. The transformants were further confirmed by their ability to grow on replica and PCR. In Fig. 1, transformants were tested by multiplex PCR for the presence or absence of target genes (all are not in the SCC element) (63) and compared with the recipient strain Nef. Transformation frequency was calculated as the ratio of the number of transformants to the total CFU of the recipient after transformation. The detection limits were about 10^−8^. Since the transformation frequency could vary 1–2 orders of magnitude depending on experiments (39), we repeated the experiments independently a few times (Table 1). None detected values were assigned half the value of the detection limit to facilitate the calculation of the mean values and statistical analyses. All transformation assays for MRS isolates were conducted in at least two independent experiments.

### Genome sequencing and analysis

*S. aureus* cells were harvested from log-phase cultures and lysed by 0.1  mg/mL lysostaphin before DNA was extracted using the standard phenol–chloroform method. Genome sequencing of the transformants was carried out via an MGI DNBSEQ platform (Bioengineering Lab. Co., Ltd., Japan). Short reads were assembled *de novo* using SeqMan NGen (Lasergene 18, DNASTAR). The assembled contigs were analyzed using SCCmecFinder (40), to test the presence of integrated SCC*mec* elements in transformants (Table S5). The contigs of transformants containing SCC*mec* were aligned against recipient’s genome (N315: BA000018) and donor’s genome (COL: CP000046.1 or JCSC6670: AB425824.1) by Megalign Pro using MAUVE (Lasergene 18, DNASTAR).

### Statistics

Statistical analyses were performed by GraphPad Prism using Fisher’s exact test, or unpaired two-tailed Student’s *t*-test as indicated in figure legends. Statistical significance was performed on the log values of the transformation frequencies. *P < 0.05, **P < 0.01 were considered statistically significant. Error bars represent SD from two or more independent experiments.

## Data Availability

Sequencing data have been deposited in NCBI under BioProject ID PRJNA1283923.

## SUPPLEMENTAL MATERIAL

Supplemental Material msphere.00442-25-s0001.pdf Supplemental tables and figures.

ASM does not own the copyrights to Supplemental Material that may be linked to, or accessed through, an article. The authors have granted ASM a non-exclusive, world-wide license to publish the Supplemental Material files. Please contact the corresponding author directly for reuse.
