# Supplementary material for: Mixed-biofilm natural transformation assay reveals the presence of staphylococci in human environments that can transfer SCCmec to Staphylococcus aureus
Source: mSphere. 2025 Sep 22;10(10):e00442-25. doi: 10.1128/msphere.00442-25 (PMC12570499; doi:10.1128/msphere.00442-25)
Supplement: Supplemental Material — Supplemental tables and figures. [file msphere.00442-25-s0001.pdf]

Table S1

Table S1. Pet isolates.

mecA + strain	Isolation date	Location	Source	SCC*mec* type	Species	Inhibitory zone (mm)		Erythromycin resistance (≥16 μg/mL)	Tetracycline resistance (≥5 μg/mL)	Kanamycin resistance (≥50 μg/mL)	Chloramphenicol resisrance (≥12.5 μg/mL)
						MPI 1 μg/mL	CFX 30 μg/mL				
J-P1	2019	Japan	Pet	Unknown	*S. pseudintermedius*	7	17	R	S		
J-P2	2019	Japan	Pet	V	*S. pseudintermedius*	7	21	S			
J-P3	2022	Japan	Pet	IVa/IVc	*S. epidermidis*	11	25	S			
J-P4	2022	Japan	Pet	IVa/IVc	*S. epidermidis*	0	17	S			
J-P5	2022	Japan	Pet	Unknown	*S. epidermidis*	0	17	S			
J-P6	2022	Japan	Pet	Unknown	*S. felis*	23	31	R	S		
J-P7	2022	Japan	Pet	Unknown	*S. nepalensis*	0	18	S			
J-P8	2022	Japan	Pet	Unknown	*S. epidermidis*	0	9	S			
J-P9	2022	Japan	Pet	Unknown	*S. nepalensis*	0	9	S			
J-P10	2022	Japan	Pet	V/IVa	*S. epidermidis*	7	19	S			
J-P11	2022	Japan	Pet	III	*S. capitis*	0	0	S			
J-P12	2022	Japan	Pet	V	*S. epidermidis*	0	16	S			
J-P13	2022	Japan	Pet	III	*S. epidermidis*	0	0	R	S		
J-P14	2022	Japan	Pet	V	*S. cohnii*	0	9	R	S		
J-P15	2022	Japan	Pet	IVc/IVb	*S. felis*	0	7	S			
J-P16	2022	Japan	Pet	IVc/IVb	*S. felis*	0	11	S			
J-P17	2022	Japan	Pet	IVc/IVb	*S. felis*	0	10	S			
J-P18	2022	Japan	Pet	III	*S. capitis*	13	15	S			
J-P19	2022	Japan	Pet	IVa	*S. epidermidis*	0	15	R	S		
J-P20	2022	Japan	Pet	III	*S. cohnii*	0	14	S			
J-P21	2022	Japan	Pet	Unknown	*S. felis*	28	36	S			
J-P22	2022	Japan	Pet	Unknown	*S. lugdunensis*	7	22	S			
J-P23	2022	Japan	Pet	Unknown	*S. schleiferi*	7	16	S			
J-P24	2022	Japan	Pet	Unknown	*S. felis*	0	21	R	S		
J-P25	2022	Japan	Pet	V/IVc	*S. epidermidis*	14	21	S			
J-P26	2022	Japan	Pet	IVc	*S. warneri*	0	15	R	S		
J-P27	2022	Japan	Pet	Unknown	*S. warneri*	16	25	S			
J-P28	2022	Japan	Pet	IVc/IVb	*S. felis*	23	33	S			
J-P29	2022	Japan	Pet	IVc	*S. epidermidis*	24	29	S			
J-P30	2022	Japan	Pet	Unknown	*S. felis*	11	25	R	S		
J-P31	2022	Japan	Pet	IVb	*S. felis*	23	34	S			
J-P32	2022	Japan	Pet	Unknown	*S. lugdunensis*	0	24	S			
J-P33	2022	Japan	Pet	Unknown	*S. felis*	20	29	S			
J-P34	2023	Japan	Pet	III	*S. nepalensis*	0	11	R	S	S	S
J-P35	2023	Japan	Pet	III	*S. cohnii*	8	23	S	S	S	S
J-P36	2023	Japan	Pet	III	*S. pseudintermedius*	0	12	R	S	R	R
J-P37	2023	Japan	Pet	V	*S. xylosus*	14	25	R	S	R	R
J-P38	2023	Japan	Pet	V	*S. pettenkoferi*	16	20	S	S	S	S
J-P39	2023	Japan	Pet	III	*S. pettenkoferi*	17	18	S	S	S	S
J-P40	2023	Japan	Pet	V/IVa	*S. epidermidis*	0	12	R	S	R	R

Table S2

Table S2. Human isolates.

						Inhibitory zone (mm)					
***mecA* + strain**	**Isolation date**	**Location**	**Source**	**SCC*mec* type**	**Species**	**MPI 1 µg/mL**	**CFX 30 µg/mL**	**Erythromycin resistance (≥16 µg/mL)**	**Tetracycline resistance (≥5 µg/mL)**	**Kanamycin resistance (≥50 µg/mL)**	**Chloramphenicol resisrance (≥12.5 µg/mL)**
J-H1	2023	Japan	Human (inpatient)	Unknown	*S. epidermidis*	0	0	R	S	R	S
J-H2	2023	Japan	Human (inpatient)	II/V	*S. epidermidis*	0	7	S	R	S	S
J-H3	2023	Japan	Human (inpatient)	III/V	*S. epidermidis*	0	11	S	R	R	S
J-H4	2023	Japan	Human (inpatient)	II	*S. caprae*	0	20	S	R	R	S
J-H5	2023	Japan	Human (inpatient)	Unknown	*S. caprae*	0	19	R	R	R	S
J-H6	2023	Japan	Human (inpatient)	I	*S. capitis*	7	15	S	R	R	S
J-H7	2023	Japan	Human (inpatient)	I/IVa	*S. epidermidis*	0	18	S	R	R	S
J-H8	2023	Japan	Human (inpatient)	I/IVa	*S. petteukoferi*	7	20	R	R	R	S
J-H9	2023	Japan	Human (inpatient)	I/IVa	*S. epidermidis*	0	16	S	S	R	S
J-H10	2023	Japan	Human (inpatient)	III/V	*S. epidermidis*	0	7	R	S	R	S
J-H11	2023	Japan	Human (inpatient)	Unknown	*S. borealis*	0	15	S	S	R	S
J-H12	2023	Japan	Human (inpatient)	III/V	*S. epidermidis*	0	15	S	S	R	R
J-H13	2023	Japan	Human (inpatient)	III	*S. caprae*	12	21	R	S	R	S
J-H14	2023	Japan	Human (inpatient)	IVc	*S. epidermidis*	0	17	R	R	R	S
J-H15	2023	Japan	Human (inpatient)	Unknown	*S. epidermidis*	11	20	S	S	R	R
J-H16	2024	Japan	Human (inpatient)	IVb	*S. capitis*	0	0	S	R	R	R
J-H17	2024	Japan	Human (inpatient)	IVb	*S. caprae*	0	19	R	S		
J-H18	2024	Japan	Human (inpatient)	V	*S. epidermidis*	0	19	S	S	R	S
J-H19	2024	Japan	Human (inpatient)	I/IVa	*S. epidermidis*	0	15	R	S	R	R
J-H20	2024	Japan	Human (inpatient)	I	*S. capitis*	0	0	S	S	R	R
J-H21	2024	Japan	Human (inpatient)	I	*S. capitis*	0	0	S	R	R	R
J-H22	2024	Japan	Human (inpatient)	I	*S. capitis*	0	0	S	R	R	S
J-H23	2017	Japan	Human (inpatient)	Unknown	*S. hominis*	0	10	R	S	R	S
J-H24	2017	Japan	Human (inpatient)	Unknown	*S. epidermidis*	0	0	R	S	R	R
J-H25	2017	Japan	Human (inpatient)	Unknown	*S. capitis*	11	17	S	S	R	S
J-H26	2017	Japan	Human (inpatient)	IVa	*S. epidermidis*	0	0	S	S	R	S
J-H27	2017	Japan	Human (inpatient)	IVa	*S. capitis*	0	17	R	S	R	S
J-H28	2017	Japan	Human (inpatient)	VI	*S. lugdunensis*	0	0	R	S	R	S
J-H29	2017	Japan	Human (inpatient)	III	*S. epidermidis*	0	0	R	S	R	R
J-H30	2017	Japan	Human (inpatient)	IVa	*S. epidermidis*	7	17	S	S	R	S
J-H31	2017	Japan	Human (inpatient)	IVa/I	*S. epidermidis*	0	8	S	S	R	R
J-H32	2017	Japan	Human (inpatient)	Unknown	*S. hominis*	0	13	R	S	R	S
J-H33	2017	Japan	Human (inpatient)	Unknown	*S. haemolyticus*	0	12	R	S	R	S
J-H34	2017	Japan	Human (inpatient)	IVa	*S. epidermidis*	0	11	R	S	R	S
J-H35	2017	Japan	Human (inpatient)	I	*S. caprae*	13	19	S	S	R	S
J-H36	2017	Japan	Human (inpatient)	IVa	*S. epidermidis*	0	10	S	S	R	R
J-H37	2017	Japan	Human (inpatient)	I	*S. capitis*	0	7	S	S	R	R

Table S3

Table S3. Meat isolates.

mecA + strain	Isolation date	Location	Source	SCC*mec* type	Species	Inhibitory zone (mm) MPI 1 µg/mL	CFX 30 µg/mL	Erythromycin resistance (≥16 µg/mL)	Tetracycline resistance (≥5 µg/mL)	Kanamycin resistance (≥50 µg/mL)	Chloramphenicol resisrance (≥12.5 µg/mL)
V-M1	2022	Vietnam	Meat (Pork)	Unknown	*S. sciuri*	0	11	S	S	S	S
V-M2	2022	Vietnam	Meat (Pork)	Unknown	*S. sciuri*	0	7	R	R	S	R
V-M3	2022	Vietnam	Meat (Pork)	Unknown	*S. sciuri*	7	18	S	R	S	R
V-M4	2022	Vietnam	Meat (Beef)	Unknown	*S. fleurettii*	0	11	S	S	S	S
V-M5	2022	Vietnam	Meat (Chicken)	Unknown	*S. sciuri*	0	15	S	R	S	S
V-M6	2022	Vietnam	Meat (Wild boar)	Unknown	*S. sciuri*	0	7	S	S	S	S
V-M7	2022	Vietnam	Meat (Wild boar)	II	*S. saprophyticus*	0	7	R	S	S	S
V-M8	2022	Vietnam	Meat (Deer)	Unknown	*S. sciuri*	0	11	S	S	R	R
V-M9	2022	Vietnam	Meat (Deer)	Unknown	*S. sciuri*	0	0	S	S	S	S
V-M10	2022	Vietnam	Meat (Deer)	Unknown	*S. sciuri*	0	12	S	S	S	R
V-M11	2022	Vietnam	Meat (Pork)	Unknown	*S. saprophyticus*	0	9	R	S	S	R
V-M12	2022	Vietnam	Meat (Pork)	Unknown	*S. haemolyticus*	0	13	S	R	R	R
V-M13	2022	Vietnam	Meat (Pork)	Unknown	*S. haemolyticus*	0	13	R	S	S	S
V-M14	2022	Vietnam	Meat (Pork)	IV	*S. haemolyticus*	14	21	R	S	R	S
V-M15	2022	Vietnam	Meat (Pork)	V	*S. haemolyticus*	7	7	S	R	R	S
V-M16	2022	Vietnam	Meat (Pork)	Unknown	*S. cohnii*	0	10	R	S	R	R
V-M17	2022	Vietnam	Meat (Pork)	Unknown	*S. saprophyticus*	0	10	R	S	R	R
V-M18	2022	Vietnam	Meat (Pork)	Unknown	*S. epidermidis*	0	12	S	R	S	R
V-M19	2022	Vietnam	Meat (Pork)	Unknown	*S. saprophyticus*	0	10	R	R	S	S
V-M20	2022	Vietnam	Meat (Pork)	III	*S. saprophyticus*	0	0	R	R	S	R
V-M21	2022	Vietnam	Meat (Pork)	Unknown	*S. sciuri*	0	13	S	R	S	R
T-M1	2022	Thailand	Meat (Pork-neck)	Unknown	*S. haemolyticus*	15	17	S	R	S	S
T-M2	2022	Thailand	Meat (Pork-neck)	Unknown	*S. sciuri*	0	15	R	R	S	R
T-M3	2022	Thailand	Meat (Pork-neck)	IVa	*S. haemolyticus*	10	16	R	R	S	R
T-M4	2022	Thailand	Meat (Pork-neck)	Unknown	*S. sciuri*	0	7	R	R	S	R
T-M5	2022	Thailand	Meat (Chicken-nasopharynx)	Unknown	*S. gallinarum*	0	10	R	R	S	R
T-M6	2022	Thailand	Meat (Chicken-nasopharynx)	III	*S. sciuri*	0	14	R	S	R	R
T-M7	2022	Thailand	Meat (Chicken)	Unknown	*S. saprophyticus*	0	12	R	R	S	S
T-M8	2022	Thailand	Meat (Chicken)	Unknown	*S. sciuri*	0	16	S	S	R	R
T-M9	2022	Thailand	Meat (Chicken-leg)	Unknown	*S. saprophyticus*	0	11	R	R	S	S
T-M10	2022	Thailand	Meat (Chicken-leg)	Unknown	*S. scuiri*	0	15	R	R	S	S
T-M11	2022	Thailand	Meat (Chicken-intestinal)	Unknown	*S. haemolyticus*	16	20	R	R	S	S
T-M12	2022	Thailand	Meat (Pork-leg)	Unknown	*S. borealis*	9	16	R	R	S	R
T-M13	2022	Thailand	Meat (Pork-leg)	Unknown	*S. haemolyticus*	10	12	R	R	S	R
T-M14	2022	Thailand	Meat (Pork-leg)	Unknown	*S. borealis*	0	7	R	R	S	R
T-M15	2022	Thailand	Meat (Chicken-leg)	Unknown	*S. warneri*	7	15	R	R	S	R
T-M16	2022	Thailand	Meat (Chicken)	Unknown	*S. gallinarum*	0	10	R	R	S	R
T-M17	2022	Thailand	Meat (Pork-leg)	Unknown	*S. haemolyticus*	9	13	R	R	S	R
T-M18	2022	Thailand	Meat (Pork-leg)	Unknown	*S. haemolyticus*	7	14	R	R	S	R
T-M19	2022	Thailand	Meat (Pork-leg)	Unknown	*S. borealis*	12	17	R	R	S	R

Table S4

Table S4. Livestock isolates.

mecA + strain	Isolation date	Location	Source	SCC*mec* type	Species	Inhibitory zone (mm)		Erythromycin resistance (≥16 μg/mL)	Tetracycline resistance (≥5 μg/mL)	Kanamycin resistance (≥100 μg/mL)	Chloramphenicol resisrance (≥12.5 μg/mL)
						MPI 1 μg/mL	CFX 30 μg/mL				
V-L1	2023	Vietnam	Livestock (Chicken-wing)	Unknown	*S. arlettae*	0	18	R	R	S	R
V-L2	2023	Vietnam	Livestock (Chicken-feces)	V	*S. arlettae*	0	12	R	R	S	R
V-L3	2023	Vietnam	Livestock (Chicken-abattoir)	V	*S. lentus*	0	23	R	R	S	R
V-L4	2023	Vietnam	Livestock (Pig-nose)	Unknown	*S. cohnii*	0	0	R	R	S	R
V-L5	2023	Vietnam	Livestock (Rice feed for swine)	Unknown	*S. sciuri*	0	15	R	R	S	R
V-L6	2023	Vietnam	Livestock (Rice feed for swine)	Unknown	*S. sciuri*	0	18	R	R	S	R
V-L7	2023	Vietnam	Livestock (Chicken-foot)	IVd	*S. gallinarum*	24	0	R	R	S	R
V-L8	2023	Vietnam	Livestock (Chicken-feces)	Unknown	*S. gallinarum*	24	11	R	R	S	R
V-L9	2023	Vietnam	Livestock (Chicken-feces)	Unknown	*S. gallinarum*	0	11	R	R	S	R
V-L10	2023	Vietnam	Livestock (Chicken-feces)	Unknown	*S. gallinarum*	23	17	S	R	R	R
V-L11	2023	Vietnam	Livestock (Farm water for chicken)	Unknown	*S. cohnii*	0	14	R	R	S	R
V-L12	2023	Vietnam	Livestock (Farm water for chicken)	Unknown	*S. arlettae*	0	14	R	R	S	R
V-L13	2023	Vietnam	Livestock (Chicken)	Unknown	*S. gallinarum*	0	9	R	R	S	R
V-L14	2023	Vietnam	Livestock (Chicken-feces)	Unknown	*S. gallinarum*	0	11	R	R	S	R
V-L15	2023	Vietnam	Livestock (Chicken-anus)	Unknown	*S. gallinarum*	29	8	R	R	S	R
V-L16	2023	Vietnam	Livestock (Chicken)	Unknown	*S. sciuri*	31	13	R	R	S	R
V-L17	2023	Vietnam	Livestock (Chicken)	Unknown	*S. gallinarum*	0	13	R	R	S	R
V-L18	2023	Vietnam	Livestock (Chicken)	Unknown	*S. gallinarum*	0	10	R	R	S	R
V-L19	2023	Vietnam	Livestock (Chicken-anus)	Unknown	*S. gallinarum*	0	8	R	R	S	R
V-L20	2023	Vietnam	Livestock (Chicken)	Unknown	*S. gallinarum*	0	11	R	R	S	R
V-L21	2023	Vietnam	Livestock (Farm water for chicken)	IVa	*S. gallinarum*	0	10	R	R	S	R
V-L22	2023	Vietnam	Livestock (Farm water for chicken)	IVa	*S. gallinarum*	0	11	R	R	S	R
V-L23	2023	Vietnam	Livestock (Farm water for chicken)	Unknown	*S. sciuri*	0	14	R	R	S	R
V-L24	2023	Vietnam	Livestock (Farm water for swine)	IVa	*S. sciuri*	0	0	R	R	S	R
V-L25	2023	Vietnam	Livestock (Pig-anus)	IVa	*S. sciuri*	0	14	S	R	R	R
V-L26	2023	Vietnam	Livestock (Duck)	Unknown	*S. cohnii*	0	12	S	R	R	R
V-L27	2023	Vietnam	Livestock (Duck)	Unknown	*S. sciuri*	23	11	R	R	S	R
V-L28	2023	Vietnam	Livestock (Duck-feces)	Unknown	*S. cohnii*	37	13	S	R	R	R
V-L29	2023	Vietnam	Livestock (Cow-feces)	IVd	*S. sciuri*	0	7	R	R	S	R
V-L30	2023	Vietnam	Livestock (Cow-feces)	Unknown	*S. sciuri*	0	10	S	R	S	R
V-L31	2023	Vietnam	Livestock (Cow-feces)	IVd	*S. gallinarum*	0	11	R	R	S	R
V-L32	2023	Vietnam	Livestock (Pig-nose)	IVd	*S. cohnii*	0	14	R	R	S	R
V-L33	2023	Vietnam	Livestock (Pig-nose)	IVa	*S. saprophyticus*	0	12	R	R	S	R
V-L34	2023	Vietnam	Livestock (Farm water for swine)	IVd	*S. cohnii*	10	17	R	R	S	R
V-L35	2023	Vietnam	Livestock (Cow)	Unknown	*S. sciuri*	0	0	R	R	S	R
V-L36	2023	Vietnam	Livestock (Cow-nose)	Unknown	*S. sciuri*	0	10	S	R	R	R
V-L37	2023	Vietnam	Livestock (Cow)	IVa	*S. sciuri*	0	9	R	R	S	R
V-L38	2023	Vietnam	Livestock (Cow-nose)	Unknown	*S. cohnii*	22	9	S	R	R	R
V-L39	2023	Vietnam	Livestock (Cow-nose)	Unknown	*S. kloosii*	10	18	R	R	S	R
V-L40	2023	Vietnam	Livestock (Cow-nose)	IVa	*S. sciuri*	0	9	S	R	R	R

Table S5

Table S5. List of *S. aureus* strains & primers used in this study

Strain	Description	Source
Nef	N315ex w/oφ (N315ex cured of the φN315 prophage), 4 erm^R genes in genome, clonal complex 5	Morikawa et al, 2012
attB*	Nef with mutated *attB* site	Maree et al., 2022
NefΔcomE	Nef lacking *comE* operon	Morikawa et al, 2012
NefΔcls2-tetR	Nef carrying chromosomal tetR gene substituting *cls2* gene	Maree et al., 2022
Nef-pRI5H	Nef carrying pRIT5H plasmid, cmR	Maree et al., 2022
Nef-PcomGgfp	Nef carrying PcomGgfp reporter, kmR	Maree et al., 2023
COLw/oφ	COL strain cured of the φL54a prophage, carrying SCCmec type I clonal complex 8	Morikawa et al, 2012
98s	MSSA clinical isolate, erm^R, clonal complex unable to categorize by multiplex PCR	Maree et al., 2024
E6	MSSA clinical isolate, erm^R, clonal complex unable to categorize by multiplex PCR	Maree et al., 2024
9s	MSSA clinical isolate, clonal complex unable to categorize by multiplex PCR	Maree et al., 2022
9sΔcomE	9s mutant lacking c*omE* operon	Maree et al., 2022
9s-ermR	MSSA clinical isolate, erm^R by transduction from Nef	Maree et al., 2024
9sΔcomE-ermR	9s lacking *comE* operon, erm^R by transduction from Nef	Maree et al., 2024
RN7170	MRSA carrying SCCmec type II.4, tetS	JARBB
MS13167	MRSA carrying SCCmec type Ivd, tetS	JARBB
P57412002	MRSA carrying SCCmec type VII, ermS	JARBB
JCSC6674	MRSA carrying SCCmec type Ivh, tetS	JARBB
JCSC6668	MRSA carrying SCCmec type IVi , ermS	JARBB
JCSC6670	MRSA carrying SCCmec type Ivj, ermS	JARBB
C10682	MRSA carrying SCCmec type VIII, tetS	JARBB
**Primer**	**Sequence**	**Source**
mecAF	GTAGTTGTCGGGTTTGGT	Morikawa et al, 2012
mecAR	GGTATCATCTTGTACCCA	Morikawa et al, 2012

References

Maree, M. et al. 2024: SCC*mec* transformation requires living donor cells in mixed biofilms. Biofilm 7, 100184. doi:10.1016/j.bioflm.2024.100184

Maree, M. et al. 2022: Natural transformation allows transfer of SCC*mec*-mediated methicillin resistance in *Staphylococcus aureus* biofilms. Nat Commun 13, 2477. doi: 10.1038/s41467-022-29877-2

Morikawa, K. et al. 2012: Expression of a cryptic secondary sigma factor gene unveils natural competence for DNA transformation in *Staphylococcus aureus*. PLoS Pathog 8, e1003003. doi:10.1371/journal.ppat.1003003.

JARBB: Japan Antimicrobial Resistant Bacterial Bank, National Institute of Infectious Diseases

Table S6

Table S6. Identifiation of SCC element in transformants

Donor strain	SCC*mec* type	Data source	SCC*mec* seq availability
JCSC6668	IVi	Genbank:AB425823.1	full SCCmec seq
MS13167 (JCSC4469)	IVd	Genbank: AB097677.1	partial SCCmec sequence
C10682	VIII	Genbank: FJ390057.1	full SCCmec seq
P57412002	VII	Not Available	Not Available
JCSC6670	IVj	Genbank: AB425824.1	full SCC*mec* seq
COL	I	Genbank: CP000046.1	full SCC*mec* seq

Sequenced transformants	SCCmecFinder top hit		
Nef[JCSC6668]	IVi	This study*	Present in multiple contigs
Nef-teR[MS13167]	IVd	This study*	Present in multiple contigs
Nef-teR[C10682]	VIII	This study*	Present in multiple contigs
Nef[P57412002]	VII	This study*	Present in multiple contigs
Nef[JCSC6670]	IVj	This study*	Present in single contig
Nef-tetR[COLw/of]	I	This study*	Present in single contig

*NCBI BioProject ID: PRJNA1283923

Fig S1

A

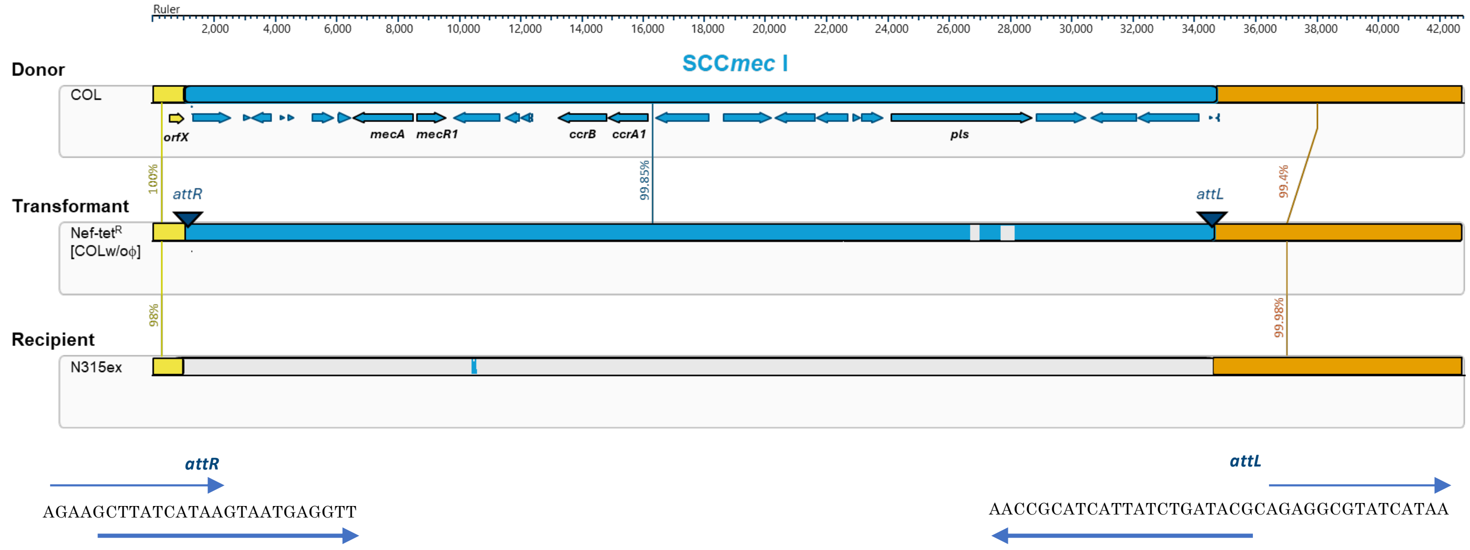

B

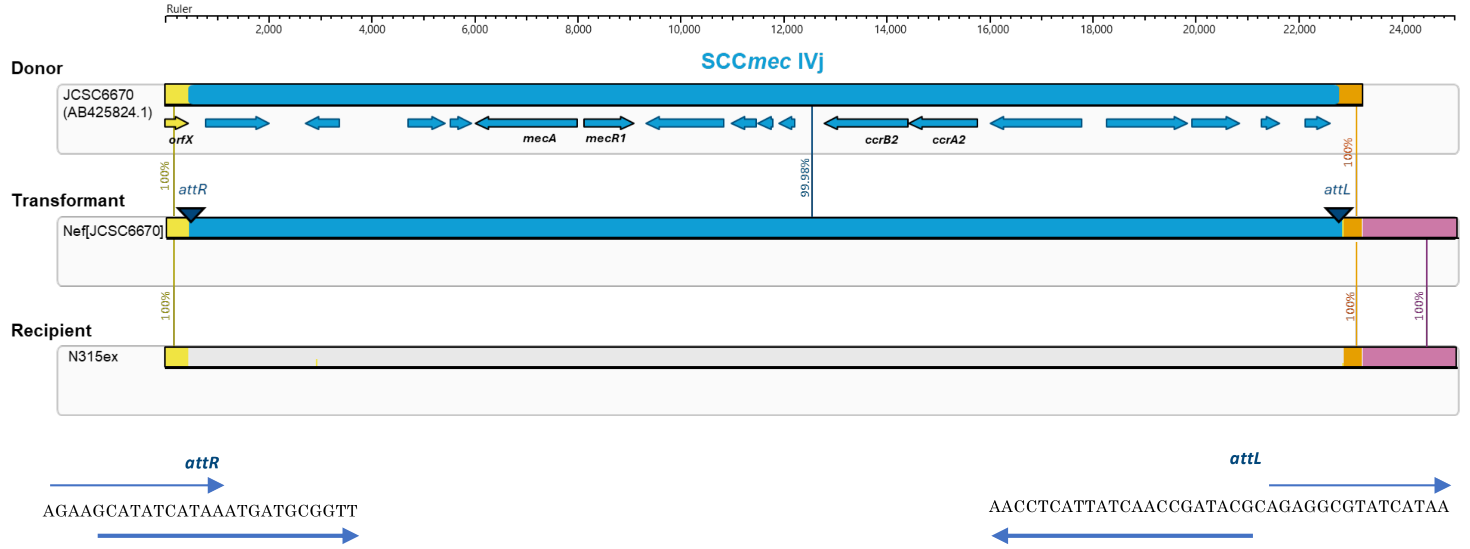

Fig. S1. Confirmation of SCC integration into recipient chromosome by genome sequencing. Assembled contigs of SCC*mec* transformants (**A**, Nef-tet^R[COLw/oϕ], **B**, Nef[JCSC6670]) were aligned with the donor genomes (COL backbone and JCSC6670, respectively; top panels) and the recipient genome (N315 backbone; bottom panels). SCC*mec* regions and associated genes are shown in blue. Homologous regions are represented by similarly colored blocks and connected by lines; grey indicates missing sequence. Percent identity of aligned regions is displayed. In both transformants, downstream regions were more similar to the recipient genome, whereas the immediate upstream region in Nef-tetR[COLw/oϕ] (highlighted in yellow) was more similar to the donor, indicating homologous recombination. Such a partial homologous recombination was also observed in our previous study (Maree et al., 2022), and this supports the fact that *attB* mutation does not abolish the SCC*mec* transformation (**Fig. 3**). A minor deletion in the *pls* gene, encoding surface protein G, was observed in Nef-tet^R[COLw/oϕ]. The *attR* and *attL* integration sites in the transformants are indicated. Chromosomal backbones of the transformants were identical to those of the recipients, as confirmed by multiplex PCR (**Fig. 1**).

Maree, M. et al. 2022: Natural transformation allows transfer of SCC*mec*-mediated methicillin resistance in *Staphylococcus aureus* biofilms. Nat Commun 13, 2477. doi: 10.1038/s41467-022-29877-2
